# Reliable change in developmental outcomes of Brain Balance^®^ participants stratified by baseline severity

**DOI:** 10.3389/fpsyg.2023.1171936

**Published:** 2023-08-22

**Authors:** Rebecca Jackson, Joshua T. Jordan

**Affiliations:** ^1^Brain Balance Achievement Centers, Naperville, IL, United States; ^2^Department of Psychology, Dominican University of California, San Rafael, CA, United States

**Keywords:** intervention, childhood, behavior, emotionality, academic, reading, motor, social

## Abstract

The effects of comprehensive multimodal programs on developmental outcomes have not been well-studied. Emerging evidence suggests a possible role for the Brain Balance^®^ (BB) program, a multimodal training program, in serving as a nonpharmacologic approach to addressing cognitive, attentional, and emotional issues in youth. In this analysis, we examined the effects of 3 months of participation in the BB program on the outcomes of children and adolescents with developmental difficulties (*N* = 4,041; aged 4–18 years; 69.7% male). Parent-rated scores on the Brain Balance–Multidomain Developmental Survey (BB-MDS) were used to assess six areas at baseline and post-program: (1) negative emotionality; (2) reading/writing difficulties; (3) hyperactive/disruptive behavior; (4) academic disengagement; (5) motor/coordination problems; and (6) social communication problems. To estimate change from pre- to post-program, we calculated effect size (Cohen’s *d*) and the Reliable Change Index (RCI) for groups stratified by baseline severity. There was a very large effect size for the moderate/high severity (*d* = 1.63) and extreme severity (*d* = 2.08) groups, and a large effect size for the mild severity group (*d* = 0.87). The average percentage of participants who observed reliable change over all BB-MDS domains was 60.1% (RCI_CTT_) for extreme severity, 46.6% (RCI_CTT_) for moderate/high severity, and 21.1% (RCI_CTT_) for baseline mild severity. In additional assessments of primitive reflexes and sensory motor activity, students demonstrated significantly diminished primitive reflexes from pre- to post-participation and significant improvements in sensory motor skills including fine motor skills, gait and aerobic ability, proprioception, rhythm and timing, and eye-gaze stability. Overall, these results demonstrate improvements in primitive reflex integration and sensory motor skills, as well as statistically significant reliable change in emotionality, reading/writing, behavior, academic engagement, motor skills, and social communication in BB participants from pre- to post-program, with the probability and degree of change increasing as the participants’ baseline severity increases. These results contribute to the growing literature on the need for evidence-based nonpharmacologic approaches to addressing developmental issues. Future research with well-controlled designs, longitudinal follow-up, implementation across settings, and participant groups in which diagnoses are known, will help to more fully characterize the effects of the BB program.

## Introduction

From infancy through adolescence, various domains of development (motor, behavioral, cognitive, social, and emotional domains) intersect, such that a deficit in one domain can negatively affect the development of another, and conversely, improvement in one area can positively influence the development of a different area (Diamond, 2000; Davis et al., 2009; Cameron et al., 2016; Hadders-Algra, 2016; Liew et al., 2018; Mancini et al., 2018). Interventions for children with developmental difficulties often focus on single domains, for example, physical therapy, nutrition interventions, sensory integration therapy, or cognitive training (Klingberg et al., 2005; Ash et al., 2017; Vaivada et al., 2017; Jeffries et al., 2019; Padmanabha et al., 2019). However, there are few published studies on the effects of comprehensive multimodal interventions or programs on developmental outcomes. Programs that comprehensively target and integrate multiple interrelated areas of development may play a critical role in supporting development and improving functional outcomes in children with developmental issues.

One such multimodal training program (Brain Balance^®^ program) has been shown in recent studies to improve cognitive performance, attentional issues, and mental well-being in children and adolescents who tested below age-appropriate levels for attention and developmental functioning prior to program participation (Jackson and Robertson, 2020; Jackson and Wild, 2021; Jackson and Jordan, 2022; Teicher et al., 2023). The rationale for the Brain Balance program is supported by an existing body of work on various types of skills training that show that functional outcomes and brain connectivity in children, adolescents, and adults can be positively altered by training and practice over time (Klingberg et al., 2005; Tang et al., 2007; Thorell et al., 2009; Oei and Patterson, 2013; Posner et al., 2015; Bediou et al., 2018; Qian et al., 2018; Sánchez-Pérez et al., 2019). The Brain Balance program involves regular frequency and duration of training, including motor skills training, sensory engagement, cognitive exercises, nutritional guidance, and academic training, along with complementary home-based exercises.

Considering the need for innovative nonpharmacological approaches that could ameliorate developmental issues in the pediatric population (such as those with ADHD), recent studies have explored the effect of the Brain Balance program in youth with baseline attentional issues. Children and adolescent (aged 4–17 years) who participated in the Brain Balance program for 3 months experienced on average a decline in ADHD symptoms in parent-rated scores on the Brown Attention-Deficit Disorder Scales® (Jackson and Jordan, 2022). More than half of these participants experienced statistically significant reliable change in attentional functioning from pre- to post-program, especially in participants who had more pronounced attentional issues at baseline. In line with these results, an open exploratory study recently found that children diagnosed with ADHD (aged 8–14 years) who underwent a combination of Brain Balance and Interactive Metronome® training for 15 weeks experienced improvement in ADHD symptoms on both parent- and clinician-rated measures, compared to typically developing controls (Teicher et al., 2023). On parental ratings, participants with ADHD had a significant reduction of 8.3 and 8.2 points on the Conner’s Parent Rating Scale Revised and on the ADHD Rating Scale IV, respectively; and on clinician ratings, training was associated with an 8.2-point reduction in total ADHD Rating Scale Scores, indicating a medium-to-large effect size (Teicher et al., 2023). Overall, this emerging evidence suggests the potential of the Brain Balance program as a nonpharmacological approach to addressing ADHD symptoms in children and adolescents.

Additional investigation on the program’s effects on cognition found that children and adolescents who participated in the Brain Balance program for 3 months showed significant overall improvements in cognitive performance, and improved performance on distinct tests of memory, reasoning, verbal ability, and concentration (Jackson and Wild, 2021). A majority of a sample of parents also reported improvements in their children’s anxiety levels and emotional functioning after 5–6 months of Brain Balance program participation (Jackson and Robertson, 2020). These findings warrant further exploration of holistic multimodal training programs in influencing the development of children and adolescents.

The aim of this study was to retrospectively review data from parent-reported surveys on outcomes in six developmental areas of Brain Balance participants (aged 4–18 years) stratified by baseline severity. Symptom severity at baseline can influence intervention outcomes in children and has been recommended to be taken into consideration during treatment-related decision making (Buitelaar et al., 1995; Zachor and Ben Itzchak, 2010; Ben Itzchak and Zachor, 2011; Reed and Osborne, 2012; Taylor et al., 2018; McElroy et al., 2019). In this study, the six areas assessed at baseline and post-program were: (1) negative emotionality; (2) reading/writing difficulties; (3) hyperactive/disruptive behavior; (4) academic disengagement; (5) motor/coordination problems; and (6) social communication problems. We used parent-rated scores on the Brain Balance–Multidomain Developmental Survey (BB-MDS), a recently validated survey that comprehensively measures multiple developmental domains and can be used in a range of ages (Jackson and Jordan, 2023). The results presented here estimate change from pre- to post-program as assessed by effect size and the percentage of Brain Balance participants that observed improvement as measured by the Reliable Change Index (RCI), for Brain Balance groups stratified by mild, moderate/high, or extreme severity at baseline. We show that the percentages of reliable change after Brain Balance participation (especially for participants with greater severity at baseline) are similar or higher than the reliable change percentages of many other single nonpharmacological interventions previously studied, such as memory training or behavioral intervention alone (Beck et al., 2010; Thoder et al., 2010).

As sensory motor skills and primitive reflexes can provide additional valuable information about a child’s development (Cameron et al., 2016; Geertsen et al., 2016; Chandradasa and Rathnayake, 2020), we also assessed sensory motor skills and atypical retention of primitive reflexes across different age groups from pre- to post-participation in the Brain Balance program (see the *Methods* section for details on these assessments). Here, we show significant improvements in five areas of sensory motor skills and integration of eight different primitive reflexes following Brain Balance participation. Taken together, the results from parent-rated BB-MDS scores along with direct sensory motor assessments of students provide evidence of improvements in developmental outcomes of students who participate in the Brain Balance program.

## Methods

### Ethical approval

Approval for this retrospective data review was granted by an institutional review board (IRB) at Advarra (Columbia, Maryland, United States), an independent organization accredited by the U.S. Office for Human Research Protections and the Association for the Accreditation of Human Research Protection Programs. The Advarra IRB determined that this retrospective data review met the requirements for exemption from IRB oversight, according to the Department of Health and Human Services regulations found at 45 CFR 46.104(d)(4). Informed parental consent was obtained for any participants prior to general enrollment in the Brain Balance program.

### Data source

We retrospectively reviewed archived BB-MDS data from parents of students enrolled at Brain Balance Achievement Center locations across the United States and who met the inclusion criteria described below. Data were derived from pre- and post-program BB-MDS data collected between 2017 and 2020 on 4,422 participants, of whom 4,041 (95.4%) provided complete cases and were included for the present study. All participants were between the ages of 4 and 18 years (Mean [*M*] = 9.91, Standard Deviation [SD] = 3.11); and 2,817 (69.7%) were male. These data have been previously used in an exploratory factor analysis to refine and validate the BB-MDS prior to its use here (Jackson and Jordan, 2023). Building on the previous validation of the BB-MDS, the use of these data in the present study went a step further to estimate change from pre- to post-participation in the Brain Balance program.

### Measures

#### Parental surveys

The BB-MDS has recently been shown to have strong measurement properties, including validated factor structure, internal reliability, and measurement invariance across age and gender (Jackson and Jordan, 2023). Parent-rated scores were collected on six BB-MDS domains, listed below. The selection of these six domains was based on: (1) previous observations from pre-enrollment Brain Balance paperwork indicating that these particular domains are the most commonly cited by parents as concerns; and (2) a body of previous research demonstrating that achievement in these domains is associated with positive developmental outcomes in school-aged children and adolescents (McClelland et al., 2000; Graziano et al., 2007; Cooper et al., 2014; Ash et al., 2017; Liew et al., 2018; Macdonald et al., 2018; Adolph and Hoch, 2020).

1. Negative emotionality (anxious, worries a lot, moody, easily experiences hurt feelings, has low self-esteem) (Graziano et al., 2007);
2. Reading/writing problems (struggles to read independently, frequently repeats/skips lines, omits small words, reverses numbers/letters while reading/writing, frequently makes spelling errors, has difficulty sounding out words) (Cooper et al., 2014);
3. Academic disengagement (lacks motivation related to school and schoolwork, work is inconsistent, frequently brings work home due to not completing assignments during school hours, does not consistently turn in completed work, makes careless mistakes and errors) (McClelland et al., 2000);
4. Hyperactive/disruptive (difficulty remaining seated for mealtime, impulsive erratic behavior, argumentative/oppositional, needs reminders to keep hands/feet/body to themselves) (Liew et al., 2018);
5. Motor/coordination problems (clumsiness, struggles with balance, awkward movements with tasks such as running, poor gross motor skills, and delayed motor milestones such as skipping and riding a bike compared to peers) (Ash et al., 2017; Liew et al., 2018; Macdonald et al., 2018; Adolph and Hoch, 2020);
6. Social communication problems (difficulty reading body language/nonverbal social cues, lacks empathy/may be insensitive to feelings of others, unaware of what others think of them, difficulty understanding humor/sarcasm, difficulty forming friendships, and poor pragmatic skills) (Cooper et al., 2014).

#### Primitive reflexes and sensorimotor activity

Before and after BB participation, students were assessed for primitive reflexes including the asymmetric tonic neck reflex, Landau reflex, Moro reflex, palmar reflex, rooting reflex, spinal galant reflex, symmetric tonic neck reflex, and tonic labyrinthine head reflex (Chandradasa and Rathnayake, 2020). Primitive reflexes were scored on a scale from 0 to 4, with 0 being no response, indicating that the reflex has been normally integrated, and a 4 being a significant response. In addition to primitive reflexes, sensory motor skills were measured in five areas as described in more detail below: (1) fine motor skills as measured by the Purdue Peg Board; (2) gait and aerobic ability as measured by the cross-crawl march and jump rope; (3) proprioception as measured by a rocker board and the one-leg balance test; (4) rhythm and timing as measured by the Interactive Metronome® (Shaffer et al., 2001); and (5) eye-gaze stability with head motion, as measured by the vestibulo-ocular reflex.

In order to assess fine motor skills, students were timed on their completion of the Purdue Peg Board using 25 pegs with the dominant hand (Squillace et al., 2015). To assess proprioception, a rocker board and the one-leg balance test was used (Fong et al., 2016; Kobel et al., 2020), where students were assessed sequentially through the following levels of difficulty until they were unable to maintain balance for the specified amount of time: (1) maintain balance with two feet on a rocker board for 30 s; (2) maintain one-leg balance on the ground with eyes open for up to 60 s; (3) maintain one-leg balance on a rocker board for up to 60 s; (4) maintain one-leg balance on the ground with eyes closed for up to 60 s; and (5) maintain one-leg balance on a rocker board with eyes closed for 60 s. Participants then repeated these levels using the other leg.

To assess gait, we used the cross-crawl march (Surburg and Eason, 1999) and jump rope (Trecroci et al., 2015), where students were assessed sequentially through the following levels of difficulty until they were no longer able to perform for the specified number of sets: (1) cross-crawl march, where students tap the hand to the opposite knee as they march, for 10 sets of marching; (2) cross-crawl march for 20 sets; (3) march while raising the opposite arm at a 90-degree angle, for 20 sets; (4) march while turning the head to the raised hand, for 20 sets; (5) cross-crawl march using a low-lateral skater pattern, for 15 sets; (6) jump rope 10 times; (7) jump rope 20 times; and (8) jump rope 40 times. Students were given verbal and visual instructions for all tasks.

### Inclusion criteria

Prior to enrolling in the Brain Balance program, prospective students were assessed at Brain Balance centers by trained technicians who had completed training in the centers’ protocols. Students who were eligible for enrollment in the Brain Balance program did not have any known genetic disorders and needed to demonstrate a developmental readiness for the program, as defined by the ability to engage with instructors and follow a one-step direction, to attempt the tasks requested, and to continue to work throughout the duration of the assessment. Re-direction and repetition of instructions both visually and verbally were allowed in the definition of readiness. Tasks were described to each student through verbal instructions as well as a physical demonstration of the task. In most instances, additional instructions beyond this initial explanation and demonstration were not needed. However, if a student did not understand the instructions, the assessor repeated the physical instructions, for example, demonstrating the placement of one peg in a pegboard at a time for the fine motor task. At the time of this assessment for students, parents completed the BB-MDS. Students who met the abovementioned inclusion criteria were then enrolled for participation in the Brain Balance program, as described in more detail in the *Training Protocol* section below. Following completion of the Brain Balance program, participants’ parents again completed the BB-MDS.

### Training protocol

Participants in the Brain Balance program attended three in-center sessions per week (for 3 months), with each session lasting 1 h (45 min of sensorimotor stimulation and 15 min of academic activities), along with other multimodal activities targeting various developmental areas (see the list below). All participants went through the same series of stations, which consisted of the following exercises and activities:

- Passive sensory stimulation in the form of tactile, olfactory, visual, and auditory stimulation (Woo et al., 2015);
- Exercises targeting primitive and postural reflexes (Chandradasa and Rathnayake, 2020), which were assigned based on indicators of a retained reflex at the time of the initial assessment. The following reflexes were assessed: Moro reflex, spinal Galant reflex, rooting reflex, palmar grasp reflex, asymmetrical tonic neck reflex, symmetrical tonic neck reflex, tonic labyrinthine reflex, and Landau reflex;
- Core muscle exercises (Myer et al., 2011);
- Proprioceptive and balance training, using a rocker board and one-leg balance (Fong et al., 2016; Kobel et al., 2020);
- Gait exercises, using the cross-crawl march (Surburg and Eason, 1999) and jump rope (Trecroci et al., 2015);
- Vestibular exercises, including rotational, translational, and anterior-to-posterior movements;
- Fine motor activities, including the palmar grasp reflex to increase muscle strength and the Purdue Peg Board to improve dexterity and speed (Squillace et al., 2015);
- Rhythm and timing exercises, including whole-body coordination activities and use of the Interactive Metronome®, a training tool that combines the concept of a musical metronome with a computerized program that measures and improves rhythm and timing (Shaffer et al., 2001);
- Activities that aim to enhance auditory and visual processing, as well as coordination and endurance of eye movements (Robert et al., 2014; Fisher et al., 2015). More specifically, auditory engagement consisted of exposure to varying levels of auditory stimulation and activities targeting the ability to filter and rapidly process auditory information. Visual stimulation was achieved through exposure to color and light stimulation, as well as exercises that require eye coordination, timing, and speed of processing perceived information.

While all participants engage with the same activity stations, each participant’s program is individualized based on their performance on the initial assessment in order to determine the appropriate starting level for each exercise. Each exercise/activity was progressive in nature and changed in duration, quantity, and complexity as the participants’ functional abilities improved over the course of the program. The criteria for making changes in the duration, quantity, and complexity of the program were based on a student achieving mastery of each level. We defined mastery as the student successfully completing the task over three consecutive sessions. For example, during the sensory stimulation component of the program, if a student cooperatively wears the tactile sensory gear for three consecutive sessions, tactile vibration will be increased to the higher increment of stronger vibration for the next session. Similarly, for fine motor tasks such as the Purdue Peg Board, once the student achieves mastery by reaching their current goal target for three consecutive sessions, the next session’s goal will be increased to place additional pegs into the pegboard holes in the same amount of time.

The academic component of the 1-h session was based on the initial functional assessment and focused on improving literacy and listening skills. During the initial assessment, each student completes a reading fluency benchmark to determine the appropriate reading level. Students whose reading level is at a PreK- to third-grade level will engage in reading fluency and comprehension activities specific to their current level of ability. Students whose reading level is at a fourth-grade level or higher will engage in a listening comprehension curriculum in which the student listens to a passage and answers questions in either details or main idea topics. If a student is struggling to read at the appropriate grade level, they will listen to the passage while reading, with each word lighting up as it is being read, to engage both visual and auditory pathways simultaneously.

In addition to the abovementioned in-center activities, parents were asked to assist their children in completing daily exercises at home and were also given nutritional guidance and support throughout the duration of the program. The home exercises consisted of 0–8 primitive reflexes (assigned if the primitive reflex was present at the time of assessment), physical fitness activities (push-ups and sit-ups), and eye strengthening exercises. To ensure consistency in parental implementation of the at-home portions of the program, parents received in-center training on how to perform the home exercises and were provided access to an online parent portal that included videos on each of the exercises as well as written instructions with photos. Any verbal and written instructions given to parents are provided in plain language that is short and simple, as education levels may vary among the participants’ parents.

### Analysis

#### Reliable change analyses for BB-MDS scores

Due to the large sample size of the study, we did not rely on tests of statistical significance to determine whether scores differed from pre- to post-program, as a negligible effect size of *d* = 0.05 is considered statistically significant, even with a stringent adjustment to correct for Type I error. We considered a multivariate analysis but chose not to use this approach as we wanted to consider change in each domain individually. Instead, we rely here on Cohen’s *d* and its associated 95% confidence intervals, and the percentage of participants that observed improvement as assessed by the Reliable Change Index (RCI) (Jacobson and Truax, 1991). Here, two approaches for the RCI were used. The first was based on the traditional RCI, which is based in classical test theory (hereafter referred to as RCI_CTT_). The RCI_CTT_ is defined as:


$$RCI_{CTT}=\frac{Post_{Raw}-Pre_{Raw}}{\sqrt{2*SE_{Pre}}}$$

Post_Raw_ and Pre_Raw_ refer to simple summary scores of the pre- and post-program data, and SE_Pre_ refers to the standard error of measurement at pretest. The RCI_CTT_ assumes equal weighting of items, such that all items are equal contributors in the estimate of the latent trait θ and that there is equal measurement precision (i.e., that reliability and the subsequent SE is the same across all levels of θ). Because the population-level SE used in RCI_CTT_ is the average of the individual SEs, which can vary across individuals, this may result in an SE that overestimates measurement precision in the middle of the distribution and underestimates measurement precision in the tails of the distribution (Jabrayilov et al., 2016). It has recently been proposed that RCI should also be estimated through Item Response Theory (IRT), as it may allow for greater precision and may be more sensitive to change than RCI_CTT_ (Jabrayilov et al., 2016). IRT does not assume that all items are equivalent indicators of θ and that the SE is not consistent across all values of θ (Embretson and Reise, 2000). The RCI_IRT_ is defined as:


$$RCI_{IRT}=\frac{{\hat{\theta }}_{Post}\;\;-\;\;\;{\hat{\theta }}_{Pre}}{\sqrt{SE{\left({\hat{\theta }}_{Pre}\right)}^{2}\;\;+\;\;\;SE{\left({\hat{\theta }}_{Post}\right)}^{2}}}$$

Where ${\hat{\theta }}_{Post}$ and ${\hat{\theta }}_{Pre}$ refer to estimates of each participant’s predicted latent trait, θ, and SE refers to the standard error of measurement based on IRT (Jabrayilov et al., 2016). Here, Graded Response Models (GRM) with Weighted Likelihood Estimation (WLE) were used to derive θ and SE(θ); there is evidence that WLE is less biased and more precise than other estimates of θ (Wang and Wang, 2001). For both RCI_CTT_ and RCI_IRT_, a cutoff value of ≤ −1.96 was used to indicate that a participant observed reliable, statistically significant change (i.e., “improved”), participants who obtained an RCI ≥ 1.96 were considered to have observed “deterioration,” and participants who obtained an RCI between −1.96 and 1.96 were considered to have experienced “no change,” as change was too small to be considered statistically significant (Jacobson and Truax, 1991). We did not attempt to estimate “clinically significant change” (Jacobson and Truax, 1991), as “functional” ranges of the BB-MDS have not been established to date.

We calculated Cohen’s *d*, RCI_CTT_, and RCI_IRT_ based on levels of baseline severity. This was done by stratifying the sample based on ${\hat{\theta }}_{Pre}$, as predicted values represent standardized estimates of the participants’ latent trait, where ${\hat{\theta }}_{Pre}$ = 0 indicates that a participant’s predicted latent trait is precisely average; ${\hat{\theta }}_{Pre}$ = 1.0 indicates a participant’s latent trait is one standard deviation above the mean, and so on. Participants were considered to be in the “mild severity” range if ${\hat{\theta }}_{Pre}$ was between 0.0 and 1.0, “moderate severity” if ${\hat{\theta }}_{Pre}$ was between 1.0 and 2.0, and in the “extreme severity” range if ${\hat{\theta }}_{Pre}$ was ≥2.0. Cohen’s Kappa (κ) was used to examine the agreement between RCI_CTT_ and RCI_IRT_.

All analyses were conducted in R (R Core Team, 2020) via the mirt (Chalmers, 2012), psych (Revelle, 2021), effsize (Torchiano, 2016), and JTRCI package (Kruijt, 2021).

#### Analyses of sensorimotor activity and primitive reflexes

All analyses were performed using SAS (9.4, Cary, NC). Age variables were calculated by subtracting the whole number of years that had passed since birth until the date the students were enrolled into the Brain Balance program. Students were placed into age groups 6–10, 11–13, and 14–17 years, and only students who had complete data from pre- and post-program were included in the analysis. Sensory motor activity levels were analyzed by creating a variable that represented the difference in levels between pre- and post-program data and was accomplished by subtracting pre-program levels from post-program levels.

Primitive reflex activity was analyzed by calculating the percentage change from pre- to post-program and was accomplished by utilizing the following formula: [(postprogram reflex – preprogram reflex) ÷ postprogram reflex × 100] and percentages were rounded to the nearest whole number. For both sensory motor and primitive reflex analyses, Wilcoxon Signed Rank Sum tests were performed because the pre- and post-program data are dependent and the data are not normally distributed. The Wilcoxon Signed Rank Sum test analyzes whether means by group differ and statistical significance may be achieved by observing a *p* value less than 0.05.

Multivariate analysis of variance (MANOVA) was performed to determine whether age or gender significantly affected outcomes collectively of dependent variables.

## Results

### Classical test and item response theory estimates of reliability

All BB-MDS domains were considered appropriate for IRT analysis, as all subscales were considered to be unidimensional via Principal Axis Factoring (the first Eigenvalue for each subscale >2.0) (Cordier et al., 2017), an examination of residual matrices suggested that items within all subscales were locally independent, there was evidence of monotonicity for all subscales, and all items fit their respective subscales well (Root Mean Square Error of Approximation S-X^2^ ≤ 0.010). Test information functions (TIF) for all BB-MDS subscales were high across three standard deviations of θ (all TIF ≥ 23.93); an examination of TIF plots suggested that each of the subscales of the BB-MDS provide most information at average to above-average values of θ (see Supplementary Figure 1). Internal consistency was “good” as assessed by CTT (Cronbach’s α) and IRT ($r_{xx}(\theta$)) for each of the subscales (Negative Emotionality: α = 0.78 and $r_{xx}(\theta$) = 0.84; Reading/Writing Difficulties: α = 0.84 and $r_{xx}(\theta$) = 0.85; Academic Disengagement: α = 0.83 and $r_{xx}(\theta$) = 0.85; Hyperactive/Disruptive: α = 0.84 and $r_{xx}(\theta$) = 0.84; Motor/Coordination Problems: α = 0.80 and $r_{xx}(\theta$) = 0.80; Social Communication Problems: α = 0.84 and $r_{xx}(\theta$) = 0.85). Reliability estimates via IRT suggest that BB-MDS subscales tend to be most reliable when θ ≥ −1.0, although several subscales demonstrated weaker reliability at very high levels of θ (i.e., Reading/Writing Difficulties, Hyperactive/Disruptive, Academic Disengagement θ ≥ 2.0; see Supplementary Figure 2).

### Effect size estimates of change pre- to post-program

**Figure 1:**
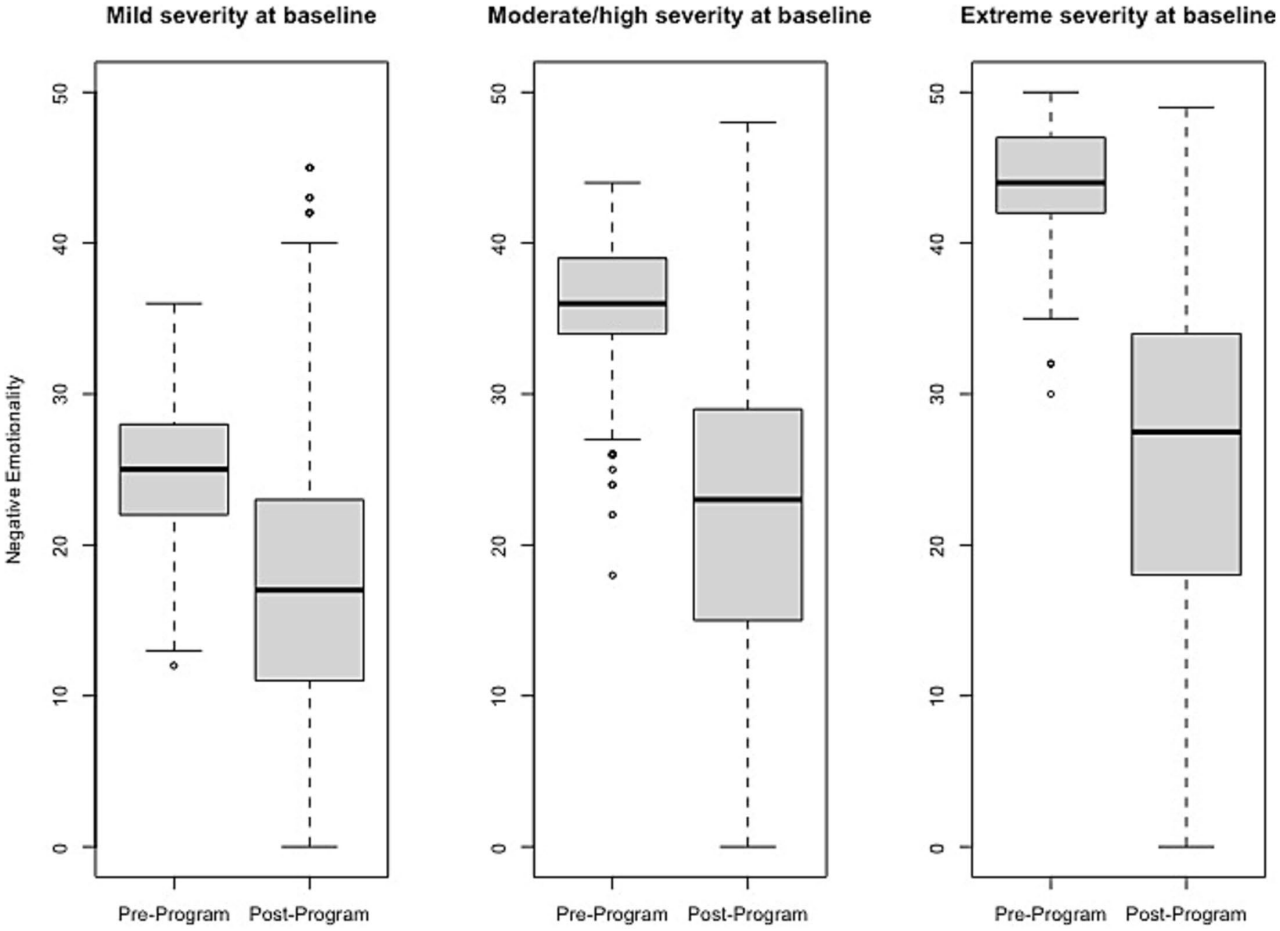
Change in Negative Emotionality raw scores, stratified by baseline severity. Shown are average pre- to post-program scores for the Negative Emotionality domain of the Brain Balance–Multidomain Developmental Survey (BB-MDS). Baseline severity is defined as theta of 0.0 to 1.0 for “mild,” 1.0 to 2.0 for “moderate/high,” and greater than 2.0 for “extreme”.

**Figure 2:**
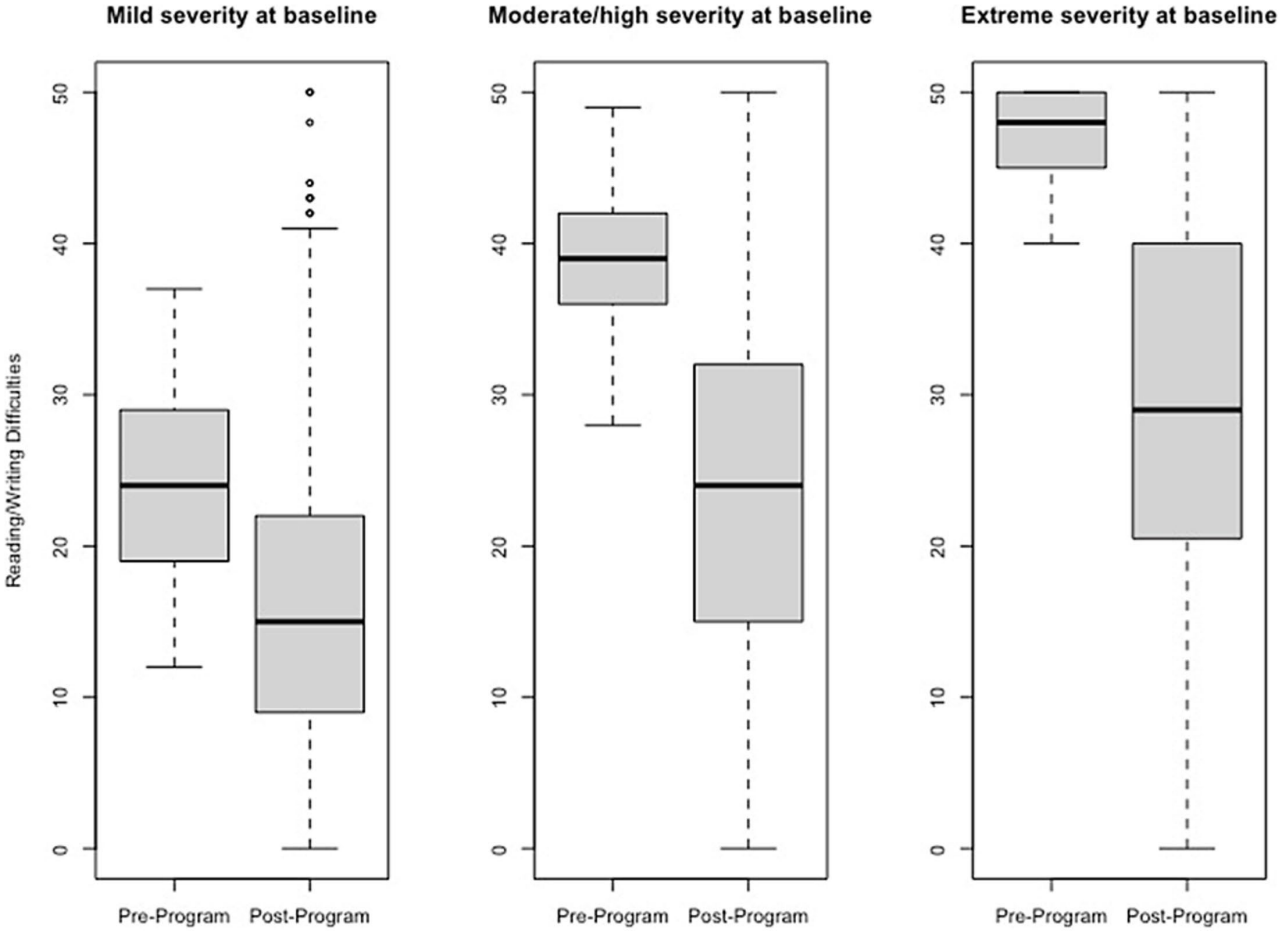
Change in Reading/Writing Difficulties raw scores, stratified by baseline severity. Shown are average pre- to post-program scores for the Reading/Writing Difficulties domain of the Brain Balance–Multidomain Developmental Survey (BB-MDS). Baseline severity is defined as theta of 0.0 to 1.0 for “mild,” 1.0 to 2.0 for “moderate/high,” and greater than 2.0 for “extreme”.

**Figure 3:**
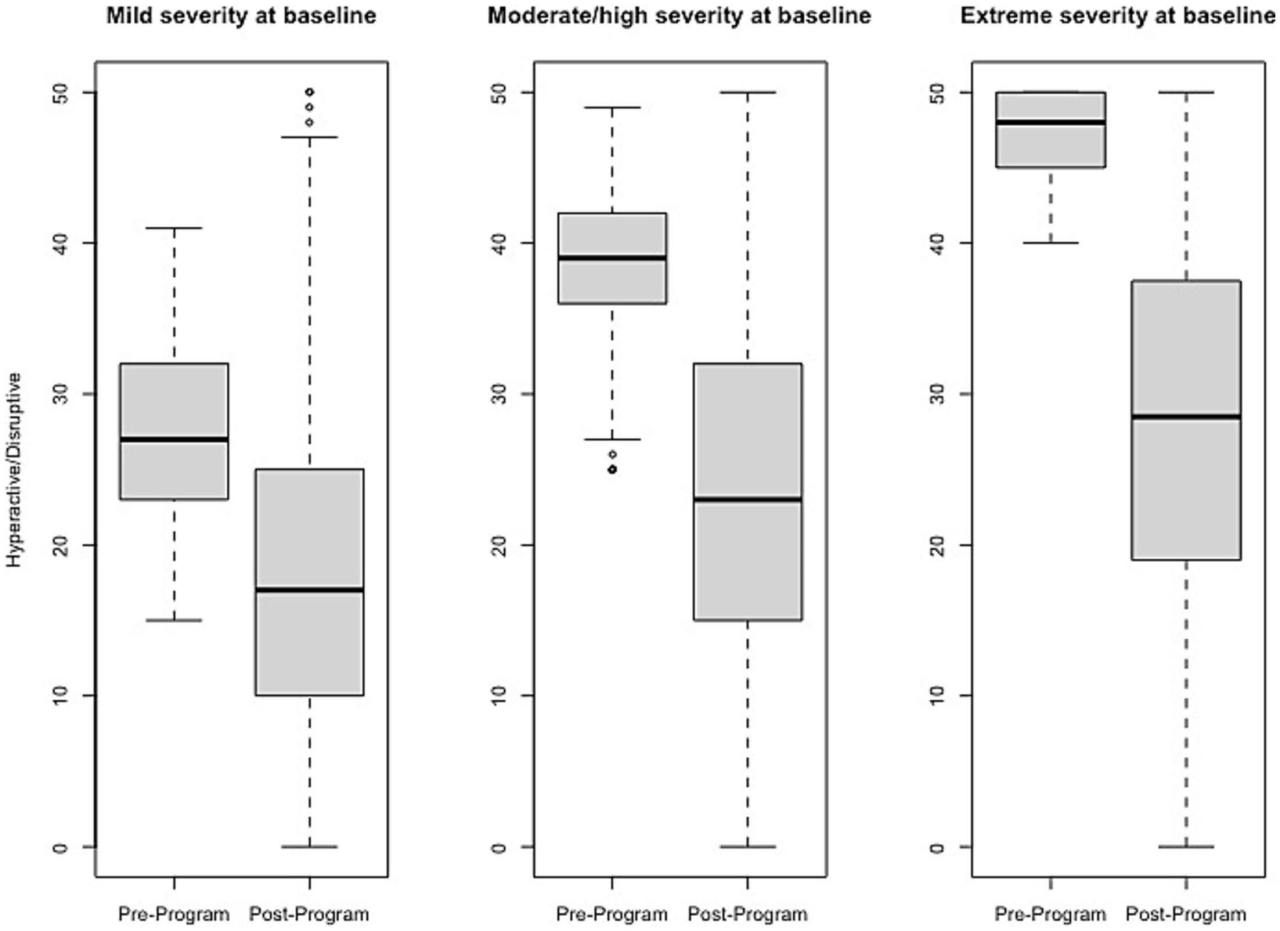
Change in Hyperactive/Disruptive raw scores, stratified by baseline severity. Shown are average pre- to post-program scores for the Hyperactive/Disruptive domain of the Brain Balance–Multidomain Developmental Survey (BB-MDS). Baseline severity is defined as theta of 0.0 to 1.0 for “mild,” 1.0 to 2.0 for “moderate/high,” and greater than 2.0 for “extreme”.

**Figure 4:**
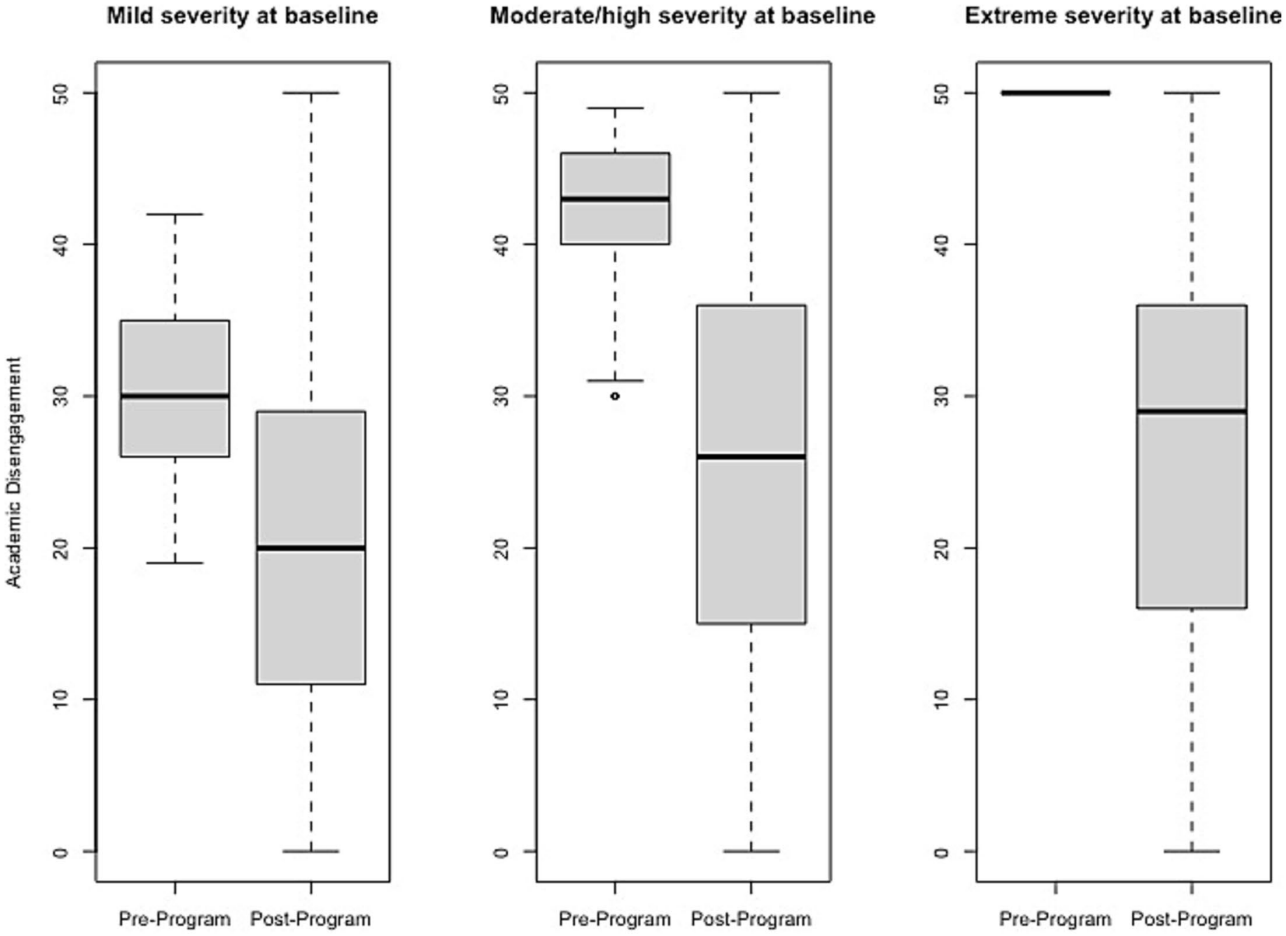
Change in Academic Disengagement raw scores, stratified by baseline severity. Shown are average pre- to post-program scores for the Academic Disengagement domain of the Brain Balance–Multidomain Developmental Survey (BB-MDS). Baseline severity is defined as theta of 0.0 to 1.0 for “mild,” 1.0 to 2.0 for “moderate/high,” and greater than 2.0 for “extreme”.

**Figure 5:**
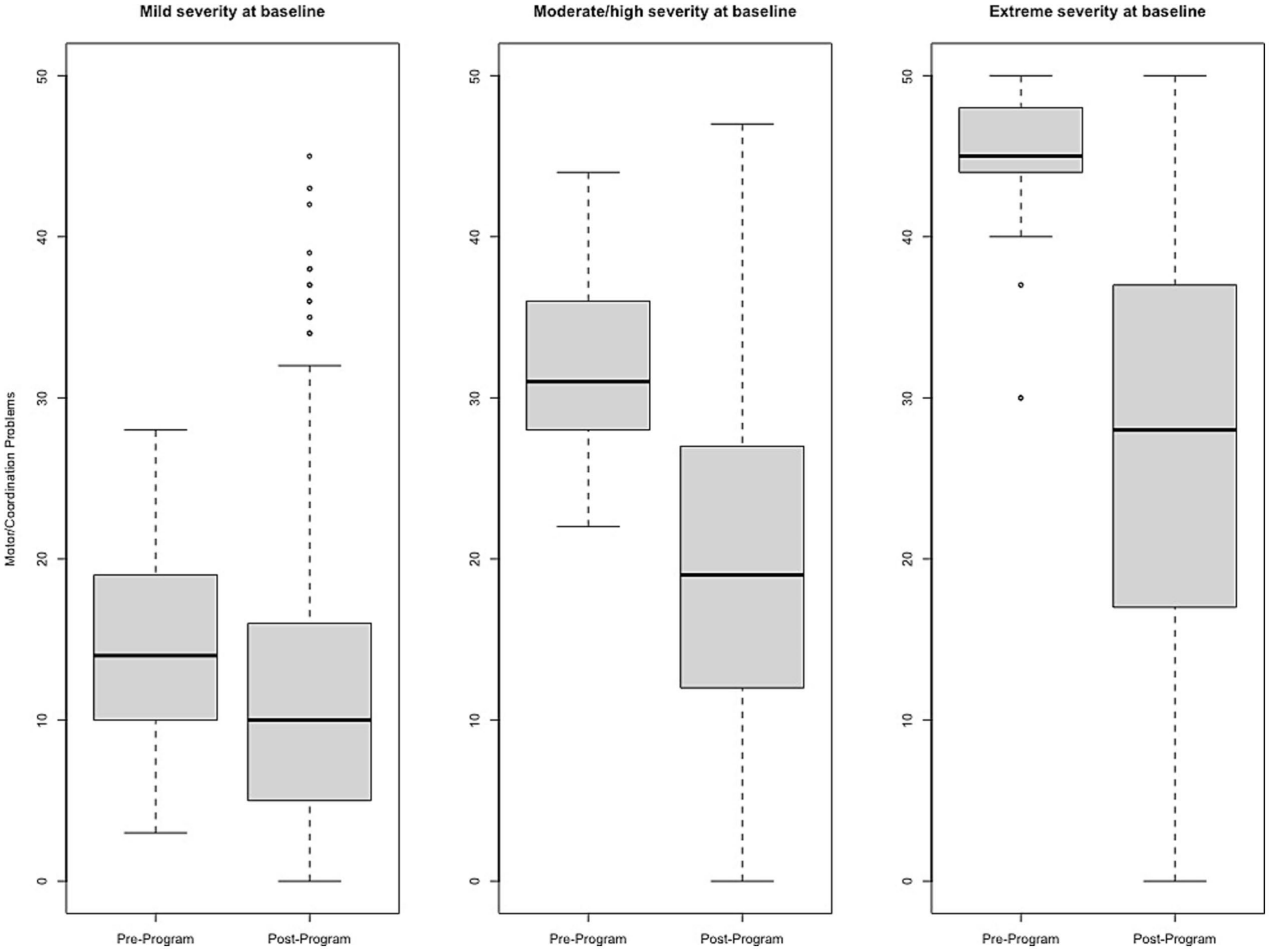
Change in Motor/Coordination Problems raw scores, stratified by baseline severity. Shown are average pre- to post-program scores for the Motor/Coordination Problems domain of the Brain Balance–Multidomain Developmental Survey (BB-MDS). Baseline severity is defined as theta of 0.0 to 1.0 for “mild,” 1.0 to 2.0 for “moderate/high,” and greater than 2.0 for “extreme”.

**Figure 6:**
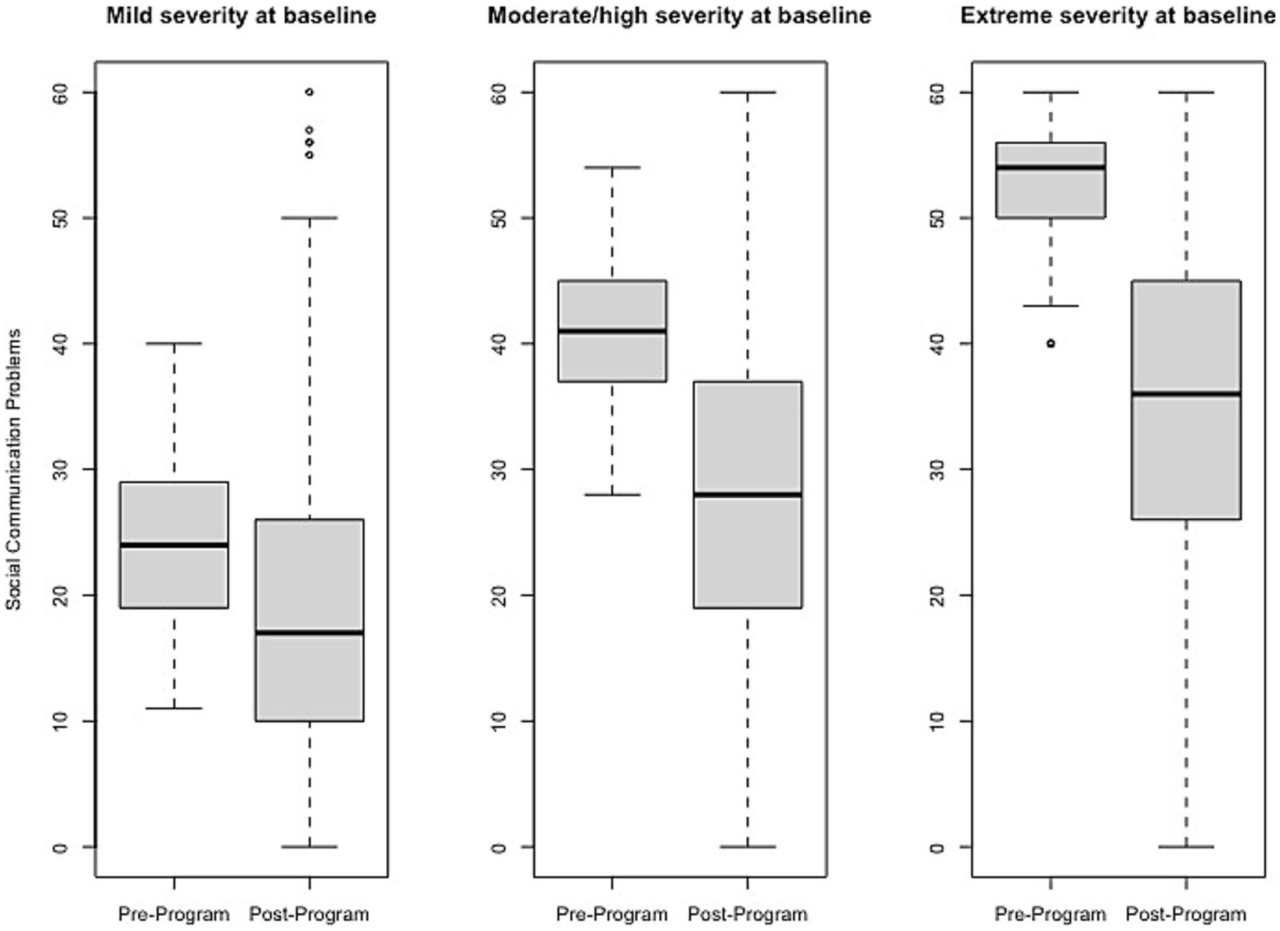
Change in Social Communication Problems raw scores, stratified by baseline severity. Shown are average pre- to post-program scores for the Social Communication Problems domain of the Brain Balance–Multidomain Developmental Survey (BB-MDS). Baseline severity is defined as theta of 0.0 to 1.0 for “mild,” 1.0 to 2.0 for “moderate/high,” and greater than 2.0 for “extreme”.

Effect size estimates based on total raw scores (Cohen’s *d* and its associated 95% confidence intervals) can be found in Table 1 and Figures 1–6 show average pre-post scores for each domain of the BB-MDS. Among participants with “mild severity” at baseline (θ = 0.0–1.0), the average effect size was “large” (*d* = 0.87); effect sizes ranged from moderate (*d* = 0.47 for Motor/Coordination problems) to very large (*d* = 1.13 for Hyperactive/Disruptive). For “moderate/high severity” participants (θ = 1.0–2.0), the average effect size was very large (*d* = 1.63); all effect sizes ranged from *d* = 1.53 to *d* = 1.80, and for participants with “extreme severity” at baseline (θ ≥ 1.0), the average effect size was *d* = 2.08, with ranges between *d* = 1.55 to *d* = 3.21.

**Table 1:** Effect size and reliable change estimates pre- to post-program.

		Reliable Change	Agreement	
Subscale	Cohen’s *d*	CTT_RCI_	IRT_RCI_	Kappa
*Baseline mild severity (*$\hat{\theta }$ *range: 0.0–1.0)*				
Negative emotionality (*n* = 1,311; 32.4%)	1.07 (0.99, 1.16)	271 (20.7%)	240 (18.3%)	0.72 (0.67, 0.76)
Reading/writing difficulties (*n* = 1,382; 34.2%)	0.88 (0.80, 0.95)	273 (19.8%)	197 (14.3%)	0.68 (0.63, 0.74)
Hyperactive/disruptive (*n* = 1,408; 34.8%)	1.13 (1.05, 1.21)	405 (28.8%)	323 (22.9%)	0.73 (0.69, 0.77)
Academic disengagement (*n* = 1,444)	1.04 (0.96, 1.12)	486 (33.7%)	331 (22.9%)	0.73 (0.69, 0.76)
Motor/Coordination Problems (*n* = 1,453)	0.47 (0.41, 0.53)	118 (8.1%)	33 (2.3%)	0.36 (0.26, 0.46)
Social communication problems (*n* = 1,366)	0.65 (0.58, 0.71)	208 (15.2%)	193 (14.1%)	0.76 (0.71, 0.81)
*Baseline moderate/high severity (*$\hat{\theta }$ *range: 1.0–2.0)*				
Negative emotionality (*n* = 617; 15.3%)	1.80 (1.64, 1.96)	288 (46.7%)	276 (44.7%)	0.85 (0.81, 0.89)
Reading/writing difficulties (*n* = 564; 14.0%)	1.65 (1.49, 1.81)	267 (47.3%)	256 (45.4%)	0.83 (0.79, 0.88)
Hyperactive/disruptive (*n* = 553; 13.7%)	1.69 (1.52, 1.85)	261 (47.2%)	294 (53.2%)	0.77 (0.72, 0.83)
Academic disengagement (*n* = 575; 14.2%)	1.74 (1.57, 1.91)	308 (53.6%)	322 (56.0%)	0.88 (0.84, 0.92)
Motor/coordination problems (*n* = 572; 14.2%)	1.53 (1.39, 1.68)	276 (48.3%)	154 (26.9%)	0.67 (0.61, 0.73)
Social communication problems (*n* = 598; 14.8%)	1.38 (1.24, 1.52)	218 (36.5%)	223 (37.3%)	0.89 (0.85, 0.93)
*Baseline extreme severity (*$\hat{\theta }$ *range: ≥ 2.0)*				
Negative emotionality (*n* = 114; 2.8%)	2.10 (1.71, 2.48)	69 (60.5%)	84 (73.7%)	0.67 (0.53, 0.81)
Reading/writing difficulties (*n* = 123; 3.0%)	1.65 (1.34, 1.96)	65 (52.8%)	76 (61.8%)	0.82 (0.72, 0.92)
Hyperactive/disruptive (*n* = 116; 2.9%)	2.06 (1.64, 2.48)	70 (60.3%)	80 (69.0%)	0.81 (0.70, 0.92)
Academic disengagement (*n* = 83; 2.1%)	3.21 (2.54, 3.88)	60 (72.3%)	67 (80.7%)	0.77 (0.61, 0.93)
Motor/coordination problems (*n* = 105; 2.6%)	1.92 (1.49, 2.35)	67 (63.8%)	66 (62.9%)	0.77 (0.65, 0.90)
Social communication problems (*n* = 110; 2.7%)	1.55 (1.22, 1.87)	56 (50.9%)	66 (60.0%)	0.78 (0.67, 0.90)

A, probability of superiority effect size; CTT, Classical Test Theory; IRT, Item Response Theory.

### Reliable change estimates from pre- to post-program

Reliable change estimates can be found in Table 1, with good agreement between RCI_CTT_ and RCI_IRT_. The agreement was similar across levels of severity, with the exception of Motor/Coordination problems for participants with “mild” severity. Per RCI_CTT_, the average percentage of participants who observed reliable change in the “mild severity” range was 21.1% (range = 8.1 to 33.7%); per RCI_IRT_, the average percentage was 15.8% (range = 2.3 to 22.9%). Among participants in the “moderate severity” range, the average percentage of participants who observed reliable change was 46.6% (range = 36.5 to 53.6%) per RCI_CTT_; using RCI_IRT_, the average percentage was 43.9% (range = 26.9 to 37.3%). For participants in the “extremely severe” range, the average percentage of participants who observed reliable change was 60.1% per RCI_CTT_ (range = 50.9 to 72.3%) and 68% per RCI_IRT_ (range = 60.0 to 80.7%). As anticipated, the probability of reliable change increased as baseline severity increased; RCI_CTT_ estimates suggest more favorable outcomes among Brain Balance participants with milder baseline symptoms than RCI_IRT_; conversely, RCI_IRT_ suggests more favorable outcomes among Brain Balance participants with more severe baseline symptoms than RCI_CTT_.

### Sensory motor activity and primitive reflexes from pre- to post-program

MANOVA showed that age significantly affected the outcome variables collectively (Wilks’ Lambda *p* < 0.0001), while gender had no effect (Wilks’ Lambda *p* < 0.41). After excluding students that did not meet the inclusion criteria, there were 6,150 students included in the analysis: 627 students aged 6–10 years, 2,356 students aged 11–13 years, and 3,167 students aged 14–17 years. The total number of students assessed for eligibility was 68,244, with 59,975 students excluded for incomplete data and 2,119 excluded for not meeting the age requirement of 6 to 18 years old. Table 2 shows mean differences observed by age group across all sensory motor metrics of interest including the Interactive Metronome, gait/aerobic, proprioception, fine motor (preferred hand), and the vestibulo-ocular reflex. Statistical significance was achieved across all metrics and age groups, with the exception of the Interactive Metronome in the 6–10 years age group (mean change 0.45; *p* < 0.09).

**Table 2:** Wilcoxon signed rank test analysis for differences in sensory motor activity level means from pre- to post-program by age group.

	6–10 Years Old	11–13 Years Old	14–17 Years Old
Sensory motor metric	Mean difference (*p* value)		
Interactive Metronome	0.45 (0.09)	3.32 (<0.0001)	7.75 (<0.0001)
Gait/aerobic	3.35 (<0.0001)	4.67 (<0.0001)	6.02 (<0.0001)
Proprioception	1.57 (<0.0001)	2.25 (<0.0001)	3.21 (<0.0001)
Fine motor (preferred hand)	2.14 (<0.0001)	3.37 (<0.0001)	4.62 (<0.0001)
Vestibulo-ocular reflex	6.01 (<0.0001)	6.71 (<0.0001)	5.86 (<0.0001)

Primitive reflex metrics included assessments of Landau, symmetrical tonic neck reflex, tonic labyrinthine reflex (head up), tonic labyrinthine reflex (head down), asymmetrical tonic neck reflex, spinal galant, rooting, Moro, and palmer reflex. Across all age groups for the primitive reflex analysis, mean changes in percentage from 35.8 to 72.5% were observed and statistical significance was achieved across all metrics and all age groups (all *p* values <0.0001), as shown in Table 3.

**Table 3:** Wilcoxon signed rank test analysis for changes in primitive reflex means pre- and post-program by age group.

	6–10 Years Old	11–13 Years Old	14–17 Years Old
Primitive reflex	Mean percentage change (*p* value)		
Landau reflex	53.5 (<0.0001)	63.7 (<0.0001)	69.9 (<0.0001)
Symmetrical tonic neck reflex	47.4 (<0.0001)	57.2 (<0.0001)	65.1 (<0.0001)
Tonic labyrinthine reflex (head up)	69.8 (<0.0001)	72.5 (<0.0001)	58.7 (<0.0001)
Tonic labyrinthine reflex (head down)	37.3 (<0.0001)	47.2 (<0.0001)	54.9 (<0.0001)
Asymmetrical tonic neck reflex	35.5 (<0.0001)	45.6 (<0.0001)	49.3 (<0.0001)
Spinal galant reflex	41.0 (<0.0001)	54.1 (<0.0001)	57.7 (<0.0001)
Rooting reflex	45.7 (<0.0001)	54.4 (<0.0001)	59.5 (<0.0001)
Moro reflex	35.8 (<0.0001)	46.3 (<0.0001)	55.4 (<0.0001)
Palmar reflex	37.6 (<0.0001)	43.8 (<0.0001)	50.5 (<0.0001)

## Discussion

Recent emerging evidence points to the role that the Brain Balance program, a holistic multimodal training program, could play as a nonpharmacologic approach to addressing cognitive (Jackson and Wild, 2021), attentional (Jackson and Jordan, 2022; Teicher et al., 2023), and emotional (Jackson and Robertson, 2020) issues in children and adolescents with developmental difficulties. The present study extends these findings by examining the effects of Brain Balance program participation on a wider range of developmental outcomes, as measured by a validated multidomain developmental survey (the BB-MDS) completed by parents of all participants before and after Brain Balance training, as well as direct pre- and post-program assessments of students in sensory motor skills and primitive reflex retention. BB-MDS data for participants stratified by baseline severity showed that the greater the severity of presenting problems at baseline, the larger the effect sizes of pre- to post-training changes and percentage of participants achieving reliable change. Participants also showed significant improvements in sensory motor skills and primitive reflex integration. These results provide evidence for the effectiveness of the Brain Balance program in improving functional outcomes in children and adolescents with developmental difficulties, especially for those who have more severe pre-existing issues.

All participants were stratified into subgroups based on the severity of their problems at baseline, in order to ascertain a clearer picture of the type of participants benefiting most from the program. As shown by analysis of parent-rated BB-MDS scores, the effect sizes increased with increasing severity — large effect size for participants presenting with mild severity at baseline, very large for participants with moderate/high severity, and even larger for those in the extreme severity group. A similar pattern was observed for the percentage of participants meeting criteria for reliable change — the average percentage of participants who observed reliable change over all BB-MDS domains increased from approximately 16–21% for baseline mild severity, to 44–47% for moderate/high severity, and reached the highest (60–68%) for Brain Balance participants in the extreme severity group. Taken together, these estimates suggest that Brain Balance participants begin to observe moderate to large effects with mild difficulties, and larger differences from pre- to post-program with increasing levels of severity.

Although many studies on developmental outcomes often do not stratify children by degree of baseline severity, there are many that group participants by baseline diagnosis (e.g., ADHD, autism spectrum disorder, developmental coordination disorder) and use RCI to evaluate changes in functioning from baseline to post-intervention. For example, previous studies have shown that children with an ADHD diagnosis who participate in non-pharmacologic behavioral interventions show varying degrees of reliable improvement from pre- to post-intervention in at least one area of behavioral or emotional functioning, with percentages of reliable change varying from 50 to 75% (Thoder et al., 2010; Rosen et al., 2019; Bayo-Tallón et al., 2020), to as low as 10% (Beck et al., 2010). Other studies of children and adolescents with diagnoses of mental health disorders such as anxiety or depression show reliable change ranging from 17 to 54% following participation in youth mental health services and psychotherapies (Arons et al., 2002; Ash and Weis, 2009; Wolpert et al., 2015; Cross et al., 2018; Boon et al., 2019; McElroy et al., 2019; Duncan et al., 2020; Cuijpers et al., 2021). Direct comparisons of these studies with the present Brain Balance study can be difficult because of differences in the type and duration of intervention/program, the specific outcomes measured, and the lack of stratification of children by degree of baseline severity. However, our percentages of reliable change for Brain Balance participants are similar or higher than many of the abovementioned non-pharmacologic interventions/programs reported on in the literature. This is particularly true for Brain Balance participants in the extreme severity group, of whom 60–68% met criteria for reliable change from pre- to post-program.

Our findings of pre- to post-program changes in two specific BB-MDS domains relating to academic functioning, namely, reading/writing difficulties and academic disengagement, could be attributed to the individualized support provided by the Brain Balance program’s academic component and/or to the program’s effects on cognitive performance and attentional functioning (Jackson and Wild, 2021; Jackson and Jordan, 2022; Teicher et al., 2023). In a previous study, Brain Balance participants displayed a significant improvement in cognitive performance, including in concentration, memory, reasoning, and verbal ability, compared to controls (Jackson and Wild, 2021). In another recent study, half of Brain Balance participants achieved reliable change in ADHD symptoms as indicated by a significant decrease in parent-rated Brown Attention-Deficit Disorder Scales® scores from pre- to post-program, especially for participants who were younger or had more pronounced attentional issues at baseline (Jackson and Jordan, 2022). An initial open study recently reported that integrated Brain Balance/Interactive Metronome training had beneficial effects on ADHD symptoms, according to parent and clinician ratings of children with ADHD (Teicher et al., 2023). In all three of these studies on cognitive and attentional functioning, beneficial effects were reported after 12–15 weeks of participation in the Brain Balance program, which is the same amount of time that participants spent in the Brain Balance program in the present study. Cognitive difficulties during childhood predict lower performance on both teacher-rated academic performance and on exam scores in reading, math, and other academic subjects (Salla et al., 2016; Lundervold et al., 2017), and specific cognitive functions such as sustained attention, working memory, and processing speed have all been associated with better performance in reading comprehension, writing, and math (Bull et al., 2008; Taub et al., 2008; Geertsen et al., 2016; Hajovsky et al., 2018). Given these findings, the changes found in the present study in reading/writing problems and academic disengagement following participation in the Brain Balance program could potentially be mediated by the program’s positive effects on cognitive and attentional functioning.

In the present study, Brain Balance participants demonstrated a significant decrease in the level of primitive reflex activity, as indicated by tests of eight different primitive reflexes across age groups ranging from 6 to 17 years old. In previous studies, significantly higher levels of primitive reflex retention have been found in school-aged children with ADHD symptoms compared with typically developing peers (Taylor et al., 2004; Konicarova et al., 2013; Bob et al., 2021; Sigafoos et al., 2021). Atypical retention of primitive reflexes has also been associated with challenges in several other developmental areas, including delayed motor development (Gieysztor et al., 2018), reading deficits (McPhillips and Sheehy, 2004), and learning difficulties (Chandradasa and Rathnayake, 2020), and could be an early indicator of neurodevelopmental conditions (Teitelbaum et al., 2004; Chinello et al., 2018; Sigafoos et al., 2021). Significantly diminished primitive reflexes following participation in the Brain Balance program may stem from components of the program involving sensory stimulation and motor skill exercises, particularly those that directly target the primitive reflexes, which may be sufficient to decrease the strength of retained primitive reflexes in students with developmental challenges.

Brain Balance participants also significantly improved in all five tests of sensory motor abilities examined, including fine motor skills, proprioception, gait and aerobic skills, rhythm and timing, and eye-gaze stability. The observed improvements across multiple sensory motor domains, together with primitive reflex integration, indicate that the Brain Balance program improves the functioning of proprioceptive and vestibular feedback mechanisms crucial for the voluntary control of complex movements and behaviors (Grigg et al., 2018; Chandradasa and Rathnayake, 2020; Sigafoos et al., 2021). Together with the parent-rated BB-MDS data, these results provide evidence in favor of a multimodal approach to improving developmental outcomes in children and adolescents with developmental issues. We have included a Theory-of-Change analysis in Table 4 to better understand how the Brain Balance program might work in improving developmental outcomes in children and adolescents.

**Table 4:** Theory-of-change analysis: Brain Balance program effects on developmental outcomes.

Elements	Description
Inputs	BB training protocol (consisting of evidence-based activities) Facilities (on-site participation at US-based BB Centers) Staff (technicians trained in BB Center protocols) Materials and equipment for BB activities Funding (from BB Centers)
Activities	Under supervision of a trained BB technician, participants attend three in-center sessions per week (1 hour/week) for 3 months, consisting of multimodal activities targeting various developmental areas: Sensory stimulation; Gross and fine motor activities; Academic activities; Exercises targeting retained primitive reflexes; Rhythm and timing exercises. A secondary at-home portion of the BB program consists of nutritional guidance along with various home exercises targeting primitive reflexes, physical fitness, and eye strengthening.
Outputs	Statistically significant reliable change in parent-rated scores on the Brain Balance–Multidomain Developmental Survey (BB-MDS) from pre- to post-program, including in emotionality, reading/writing, behavior, academic engagement, motor skills, and social communication Statistically significantly diminished primitive reflexes from pre- to post-program across assessments of eight reflexes Statistically significant improvements in assessments of five sensory motor skills from pre- to post-program, including in fine motor skills, gait and aerobic ability, proprioception, rhythm and timing, and eye-gaze stability
Outcomes	Decrease in negative emotionality, reading/writing difficulties, hyperactive disruptive behavior, academic disengagement, motor/coordination problems, and social communication problems Greater integration of primitive reflexes Improvement in sensory motor skills
Impact	Overall improved developmental functioning for children and adolescents — in the areas of emotionality, behavior, academics, social communication, sensorimotor skills, and primitive reflex integration — with positive impacts in both school and home settings.
Assumptions	Technicians across BB centers will implement the training protocol with fidelity and consistency. Materials and exercises will be at the appropriate ability level for each participant. Participants will engage with the activities as intended. Parents will implement the at-home portion of the program as they are instructed. None of the program elements will have adverse effects.
Risks and challenges	Difficulty accessing BB centers for in-person participation among students from more rural areas. Financial challenges in paying BB program tuition among participants from lower-income families.

### Limitations

The results presented here derive from program participation in a center-based setting (specifically at Brain Balance Achievement Centers). The generalizability of the program’s outcomes is therefore limited until the results are demonstrated across other types of settings. Studies are currently underway to assess Brain Balance program outcomes in home- and school-based settings.

Caution should also be taken in interpreting the evaluation of the Brain Balance program relative to other programs, as this study did not compare the program with other relevant interventions such as biofeedback or cognitive-behavioral training. The present study also did not include a control group. Future studies on the Brain Balance program will need to include direct comparisons with other interventions, as well as with a control group, in order to more accurately and fully evaluate the contribution of the Brain Balance program.

Although participants in this study were stratified by baseline severity, the sample may have been fairly heterogeneous with respect to developmental diagnoses. Information about participants’ diagnoses was not available to us, as parental disclosure of private health information (including diagnoses) was not a prerequisite for enrollment. Notably, one recent study on attentional improvements after Brain Balance participation did include participants with ADHD diagnoses and known medication status (Teicher et al., 2023). It is worth noting that many of the developmental issues that we assess in this study — sensorimotor, emotional, behavioral, and academic issues — are common across many developmental conditions; therefore, multimodal training programs that target several of these areas could still have potential to result in benefits, regardless of the specific diagnosis. It would, of course, be more helpful to know which diagnostic subgroups benefit the most, and any future studies on the Brain Balance program would need to address this. We also did not have the participants’ demographic information available to us. Future studies aiming to delineate the effects of the Brain Balance program with better precision will need to include homogenous subsamples where participants’ developmental diagnoses, medication status, and demographic information are known.

Regarding the at-home portion of the Brain Balance program, parents are provided with nutritional guidance and recommendations at home. The at-home portion is a small part of the program, as most of the Brain Balance program in this study took place in-center and was run by technicians not needing parental involvement. However, to what degree families are applying these recommendations was not tracked in the present study, leading to potential variations among participants in implementation of the at-home portions of the program. Because of potential differences in education levels of our participants’ parents, any instructions to parents regarding the at-home exercises are given in plain language that is short and simple. Going forward, adherence to home-based recommendations is being probed by survey questions at the completion of the program.

### Conclusion

The present findings suggest that the Brain Balance program is helpful in improving childhood developmental outcomes, especially for more impaired participants who have the most to gain with respect to improvement in emotional, social, behavioral, motor, and academic functioning. These results contribute to the growing literature on the need for evidence-based nonpharmacologic interventions and programs for children and adolescents with developmental issues, in order to limit the total lifetime exposure to drug treatments. Future research with well-controlled designs, longitudinal follow-up, implementation across settings, and participant groups in which diagnoses are known, will help to more fully characterize the effects of holistic multimodal approaches on the outcomes of children and adolescents with developmental and attentional challenges.

## Data availability statement

The raw data supporting the conclusions of this article will be made available by the authors, without undue reservation.

## Ethics statement

The requirement of ethical approval was waived by Advarra for the studies involving humans because the Advarra IRB determined that this retrospective data review met the requirements for exemption from IRB oversight, according to the Department of Health and Human Services regulations found at 45 CFR 46.104(d)(4). The studies were conducted in accordance with the local legislation and institutional requirements. Written informed consent for participation was not required from the participants or the participants’ legal guardians/next of kin because this study was approved as a retroactive study and met the requirements for exemption.

## Author contributions

This work was carried out in collaboration between both authors. RJ designed the study, provided the Brain Balance protocol, and contributed to the manuscript. JJ performed the analyses of the study, interpretation of the data, and manuscript writing. All authors contributed to the article and approved the submitted version.

## Conflict of interest

Author RJ was employed by Brain Balance Achievement Centres.

The remaining author declares that the research was conducted in the absence of any commercial or financial relationships that could be construed as a potential conflict of interest. The authors declare that this study received funding from Brain Balance Achievement Centres to JJ. The funder had the following involvement in the study: data collection.

## Publisher’s note

All claims expressed in this article are solely those of the authors and do not necessarily represent those of their affiliated organizations, or those of the publisher, the editors and the reviewers. Any product that may be evaluated in this article, or claim that may be made by its manufacturer, is not guaranteed or endorsed by the publisher.
