# Supplementary material for: Reliable change in developmental outcomes of Brain Balance® participants stratified by baseline severity
Source: Front Psychol. 2023 Aug 22;14:1171936. doi: 10.3389/fpsyg.2023.1171936 (PMC10478577; doi:10.3389/fpsyg.2023.1171936)
Supplement: Supplementary file 1 [file Data_Sheet_1.docx]

**Supplementary Material**

**Supplemental Figure 1.** Test information functions for Brain Balance-Multidomain Developmental Survey (BB-MDS) subscales.

**
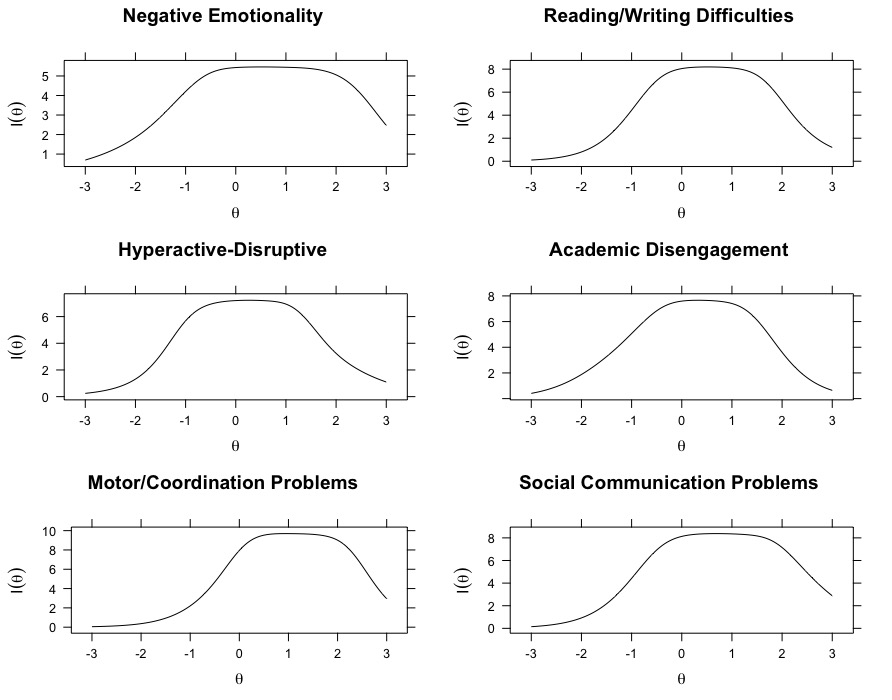
**
