# Supplementary material for: Reliable change in developmental outcomes of Brain Balance® participants stratified by baseline severity
Source: Front Psychol. 2023 Aug 22;14:1171936. doi: 10.3389/fpsyg.2023.1171936 (PMC10478577; doi:10.3389/fpsyg.2023.1171936)

**Supplemental Figure 2.** Item Response Theory (IRT)-based empirical reliability estimates for Brain Balance-Multidomain Developmental Survey (BB-MDS) subscales.


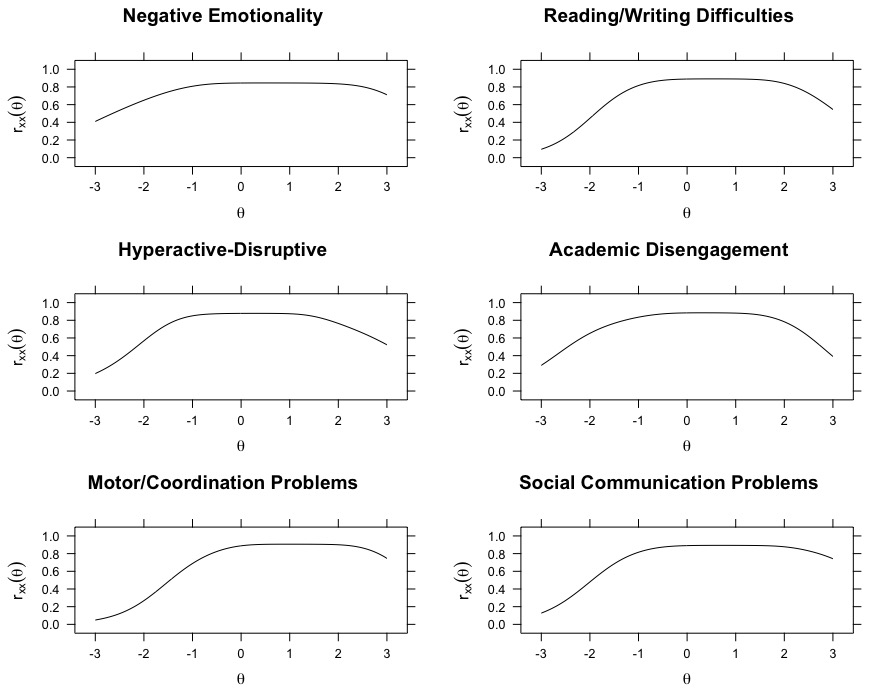

Supplement: Supplementary file 2 [file Data_Sheet_2.docx]
